# Assessing the Value of Nusinersen for Spinal Muscular Atrophy: A Comparative Analysis of Reimbursement Submission and Appraisal in European Countries

**DOI:** 10.3389/fphar.2021.750742

**Published:** 2022-01-21

**Authors:** Alessandra Blonda, Teresa Barcina Lacosta, Mondher Toumi, Steven Simoens

**Affiliations:** ^1^ Department of Pharmaceutical and Pharmacological Sciences, KU Leuven, Leuven, Belgium; ^2^ Department of Public Health, Aix-Marseille Université, Marseille, France

**Keywords:** nusinersen (spinraza), reimbursement, spinal muscular atrophy, health technology assessment (HTA), cost-effectiveness, budget impact, managed entry agreement (MEA)

## Abstract

**Background:** Nusinersen is an orphan drug intended for the treatment of spinal muscular atrophy (SMA), a severe genetic neuromuscular disorder. Considering the very high costs of orphan drugs and the expected market entry of cell and gene therapies, there is increased interest in the use of health technology assessment (HTA) for orphan drugs. This study explores the role of the economic evaluation and budget impact analysis on the reimbursement of nusinersen.

**Methods:** Appraisal reports for nusinersen were retrieved from reimbursement and HTA agencies in Belgium, Canada, France, England and Wales, Germany, Italy, Ireland, Scotland, Sweden, the Netherlands, and the United States. Detailed information was extracted on the economic evaluation, the budget impact, the overall reimbursement decision, and the managed entry agreement (MEA). Costs were adjusted for inflation and currency.

**Results:** Overall, the reports included limited data on budget impact, excluding information on the sources of data for cost and patient estimates. Only three jurisdictions reported on total budget impact, estimated between 30 and 40 million euros per year. For early-onset SMA, the incremental cost-effectiveness threshold (ICER) ranged from €464,891 to €6,399,097 per quality-adjusted life year (QALY) gained for nusinersen versus standard of care. For later-onset SMA, the ICER varied from €493,756 to €10,611,936 per QALY. Although none of the jurisdictions found nusinersen to be cost-effective, reimbursement was granted in each jurisdiction. Remarkably, only four reports included arguments in favor of reimbursement. However, the majority of the jurisdictions set up an MEA, which may have promoted a positive reimbursement decision.

**Conclusion:** There is a need for more transparency on the appraisal process and conditions included in the MEA. Additionally, by considering all relevant criteria explicitly during the appraisal process, decision-makers are in a better position to justify their allocation of funds among the rising number of orphan drugs that are coming to the market in the near future.

## Introduction

Spinal muscular atrophy (SMA) linked to chromosome 5q is a rare and life-threatening neuromuscular disorder with an estimated incidence of 1 per 12,000 births (estimated prevalence of 1–2 per 100,000 persons), making it the most frequent genetic cause of child mortality (Pearn, 1980; Verhaart et al., 2017). The disorder is characterized by a loss in alpha motor neurons in the spinal cord and brain stem, which causes progressive weakness of the proximal and respiratory muscles and motor neuron death (Castro and Iannaccone, 2014). This is a result of a deficiency of the survival motor neuron (SMN) protein, which is responsible for maintenance of these neurons. Both the SMN1 and SMN2 genes are responsible for encoding the SMN protein. In >90% of the cases, SMA is caused by a deficiency in survival motor neuron 1 (SMN1) gene, as a result of either a mutation or deletion (Lefebvre et al., 1995). The severity of the disease is thus inversely correlated by the amount of the remaining SMN2 gene copies and decreases over a spectrum from SMA type 0 to IV, with the index number relating to the maximum motor milestones achieved (see Table 1). SMA types I and II are most common, representing 87% of SMA patients (Unger, 2016).

**TABLE 1:** The different subcategories of SMA and their characteristics (Pearn, 1980; Munsat and Davies, 1992; Zerres and Rudnik-Schöneborn, 1995; Wijngaarde et al., 2020).

	SMA 0	SMA I	SMA II	SMA III	SMA IV
SMA subtype	*In utero* onset SMA	Infantile-onset SMA	Later-onset SMA	Later-onset SMA	Later-onset SMA
Symptom onset	*In utero*	Within the first 6 months of life	Between age of six and 18 months	After age of 18 months	During adulthood
Life expectancy	Within the first months of life	Within 2 years	Slightly reduced life expectancy	Normal life expectancy	Normal life expectancy
Motor milestones	No head control	Head control or rolling over to one or two sides. Not able to sit independently	Sit independently, cannot stand or walk	Stand or walk independently, trouble walking upstairs and later lose ambulation	Walk independently, mild-to-moderate muscle weakness

Due to the chronic and progressive character of SMA, patients are dependent on long-term multidisciplinary and supportive care such as orthopedic care for scoliosis and other joint deformities, gastrointestinal and nutritional care, and respiratory management, including assisted ventilation in a palliative stage (Wang et al., 2007; Crawford, 2017). Hence, SMA has a severe impact on the patient’s quality of life (QoL) and life expectancy (Landfeldt et al., 2019). Apart from the clinical burden, SMA also places a significant economic burden, in particular on parents taking care of their child with SMA (Klug et al., 2016; López-Bastida et al., 2017; Belter et al., 2020).

Nusinersen, marketed as Spinraza® by Biogen (Cambridge, MA, United States), was the first disease-modifying orphan drug indicated for the treatment of all patients with 5q SMA. It is an antisense oligonucleotide drug that promotes the expression of the SMN protein, which may lead to significant improvement in patient mobility. It is repeatedly administered intrathecally via a lumbar puncture, with patients receiving six doses within the first year and three doses within each subsequent year for the rest of their lives (Haché et al., 2016). Nusinersen was a first-in-class treatment, which addressed a large unmet need for a rare disease and was thus granted an orphan drug designation by the European Commission and the US Food and Drug Administration (FDA). It gained marketing approval from the FDA and Health Canada in 2016 and 2017, respectively, and from the European Medicines Agency (EMA) under its accelerated assessment program in 2017 (CHMP, 2017). Despite catering to the unmet needs of SMA patients, nusinersen has been criticized for its high price due to various reasons, one of which is the fact that the molecule was discovered at the University of Massachusetts, by researchers financed by CureSMA, which is a nonprofit organization that promotes research on SMA (NYTimes, 2016; Express News, 2017; Prasad, 2018; Metta, 2019; Vandekerckhove and Van Garderen, 2019; Butcher, 2019).

In order to preserve the sustainability of their healthcare systems, decision-makers across jurisdictions evaluate the cost-effectiveness and budget impact as part of a health technology (HTA) assessment. The results are then discussed during the (orphan) drug’s appraisal process, after which a decision is made regarding its reimbursement. Additionally, confidential managed entry agreements (MEAs) are set up between the payer and the pharmaceutical company, allowing reimbursement of a drug for a specified period of time, during which the company provides the treatment at a discounted price (financial-based MEAs) and/or during which additional data on real-world effectiveness may be collected in a dedicated disease or treatment registry (outcome-based MEAs). MEAs are used frequently when data on cost and/or effectiveness are scarce or uncertain, as is often the case for orphan drugs. Several studies have investigated the cost-effectiveness of nusinersen in SMA or SMA subtypes (Zuluaga-Sanchez et al., 2019; Jalali et al., 2020; Thokala et al., 2020). More recently, Dangouloff et al. (2021) performed a systematic review on the economic burden of SMA and the cost-effectiveness of its treatments, such as nusinersen (Dangouloff et al., 2021). However, it is not clear to what extent the results of cost-effectiveness and budget impact analyses played a role in the final decision-making regarding its reimbursement. Therefore, the aim of this study was to evaluate how the results of the economic evaluation and budget impact analyses, as part of the HTA, have influenced the decision on the reimbursement of nusinersen across selected jurisdictions. Additionally, we identified which jurisdictions have allowed conditional reimbursement of nusinersen by means of a MEA.

## Methods

### Search Strategy

We retrieved HTA and/or appraisal reports for nusinersen from reimbursement and HTA agencies in Belgium, Canada, France, England and Wales, Germany, Italy, Ireland, Scotland, Sweden, the Netherlands, and the US. Countries were chosen depending on the availability of public information. In addition, we have aimed to balance our country selection and reported data from countries that have adopted either a Bismarck or Beveridge model, with either a social or private security system and with an even geographical spread. Reports and relevant publications were translated via Google Translate. Information on economic evaluation was incorporated as submitted by Biogen and presented by HTA or reimbursement agencies. Additionally, in jurisdictions of which HTA reports contained no information on reimbursement decisions and MEAs, a literature search on Google Scholar or PubMed was performed to identify publications, either peer-reviewed or grey literature. These searches included combinations of keywords such as “managed entry agreement” + “France” + “nusinersen” or for instance “reimbursement” + “Italy” + “Spinraza”. We included publications between January 1, 2000, and July 20, 2020.

### Data Extraction

We created two data extraction tables that allowed a systematic data extraction from each HTA or appraisal report. Per jurisdiction, we extracted information on the economic evaluation (study design and results of the base case and sensitivity analysis) (see Table 2 and Table 3) and the budget impact analysis (see Table 4). However, not all jurisdictions are included in each table, as a result of data unavailability. For instance, the jurisdictions England and Wales were not added to Table 4 since budget impact data were not included in the HTA report. Finally, we included information on the overall reimbursement decision, the conditions for reimbursement, and the MEA in the results section.

**TABLE 2:** Overview of the design of the economic evaluation of nusinersen in six European countries, the US, and Canada.

Jurisdiction	Ireland (National Centre for Pharmacoeconomics (NCPE), 2017)	Scotland (SMC, 2018)	Sweden (NT-council, 2019)	The Netherlands (Zorginstituut Nederland, 2018; Nederland Zorginstituut, 2019; Bruins, 2019)
Perspective	-Healthcare payer perspective (base case analysis)	-Healthcare payer perspective (base case analysis)	-Societal perspective (although in its reanalysis, TLV excluded indirect costs from the basic analysis)	Societal perspective
	-Societal perspective (secondary scenario analysis)	-Societal perspective for supplementary analysis		
Economic evaluation technique	Cost-effectiveness (LYG) + cost-utility analysis (QALY)	Cost-utility analysis (QALY)	Cost-utility analysis (QALY) + cost-effectiveness analysis (LYG)	Cost-utility analysis (QALY) + cost-effectiveness analysis (LYG)
Comments of the HTA body	—	—	—	—
Economic model type	2 Markov models	2 Markov models	3 Markov models: -EO SMA type I	2 Markov models
	-EO SMA (type I)	-EO SMA (type I)	-LO SMA types II	-EO SMA (type I)
	-LO SMA (types II and III)	-LO SMA (types II and III)	-LO SMA type III	-LO SMA (type II and III)
Comments of the HTA body	—	—	LO SMA type III data, which was obtained from uncontrolled studies, was considered weak and therefore excluded from TLV’s reanalysis	-Overall, model structure was sufficient
				-Reasons for stopping therapy poorly substantiated
Comparator	Standard of care, consisting of	Standard of care, consisting of	Standard of care, consisting of	Standard of care, consisting of
	-Respiratory care	-Respiratory care	-Respiratory care	-Respiratory care
	-Gastrointestinal care	-Gastrointestinal care/nutritional care	-Gastrointestinal care	-Gastrointestinal care
	-Nutritional care	-Orthopedic care/rehabilitation care	-Nutritional care	-Nutritional care
	-Orthopedic care	-Palliative care	-Orthopedic care	-Orthopedic care
Comments of the HTA body	—	—	—	—
Time horizon	Lifetime horizon applied to both models (no further details)	Lifetime horizon	Lifetime horizon	Lifetime horizon
		-EO (SMA I): 40 years (mean initial age: 5.6 months)	-EO (SMA I): 40 years (mean initial age: 5.58 months)	-EO SMA (type I): 40 years (results measured over a period of 13 months)
		-LO (SMA II and III) 80 years (mean initial age: 43.7 months)	-LO (SMA II and III): 80 years (mean initial age: 43.71 months)	-LO SMA (type II and III): 80 years (results measured over a period of 15 months)
Comments of the HTA body	—	—	-EO SMA type I: 40-year horizon justified by ENDEAR (SMA I) data suggesting that nusinersen had a significant effect on survival	Time horizon deemed appropriate
			-LO SMA type II and III: 80-year horizon chosen according to Zerres et al. survival data	
Target population	-EO (type I) SMA patients	-EO (type I) SMA patients	-SMA I patients: < age of 6 months at diagnosis, onset of symptoms within 6 months after birth	EO (type I) SMA patient subgroup: first symptoms at age <6 months and illness duration <13 weeks at the start of treatment (data from ENDEAR trial with additional data from CS3A trial)
	-LO (type II/III) SMA patients	-LO (type II/III) SMA patients	-SMA II patients: > age of 6 months at diagnosis, onset within 6–18 months of age	LO (type II and IIIa) SMA patient subgroup: first symptoms before age of 20 months and illness duration <25 months at the start of treatment
			SMA III patients: age between 2 and 15 years at diagnosis, onset of symptoms after 18 months	
Comments of the HTA body	—	Presymptomatic SMA patients were excluded by Biogen	-Long-term number of patients to be treated is uncertain	ZIN compared patients from ENDEAR and CHERISH trial to Dutch clinical practice, based on Dutch SMA study by Wadman et al. Type I SMA correspond between ENDEAR and the Dutch study. LO SMA type II and III patients from the CHERISH trial correspond to the type IIa/b patients in Netherlands (and not type IIIa or IIIb)
			-Within the target population, it is difficult to estimate those for which nusinersen might be relevant due to comorbidities such as scoliosis surgery or mental issues	
			-Expected increase in the number of treatment-eligible patients after successful clinical practice implementation of nusinersen due to prolonged survival for SMA I and II patients	
Scope of the cost	Direct medical costs: technology and health state maintenance	Medicine acquisition, administration, SMA management, and end-of-life costs	-Direct medical cost: technology and health state maintenance	-Direct medical costs: technology and health state maintenance
			-Direct nonmedical costs: community services and traveling	-Direct nonmedical costs: (incl transport and productivity losses)
			-Indirect costs: caregiver productivity losses	-Indirect nonmedical costs (incl. productivity losses of caregiver and patient for type II and III SMA)
Comments of the HTA body	—	—	Direct costs: Uncertainty regarding costs for resources utilized for administration in their reanalysis, TLV included the indirect costs and caregiver QALYs only in the sensitivity analysis (not in the basic cost scenario)	-Appropriateness and accuracy of costs such as legal assistance, adaptations to house or car, and inclusion of productivity losses as cost components in the indirect nonmedical costs were questioned. ZIN believes the friction cost method to be more appropriate
				-Major differences in yearly cost/SMA type between studies used as a data source for cost estimates, leading to uncertainty on the methods used to define these costs
Outcomes	LYG and QALY gain, incl. patient and caregiver QALYs	LYG and QALY gain, incl. patient and caregiver QALYs	LYG and QALY gains, inclusion of caregiver disutilities	LYG and QALY gains
	**Calculation of utilities:**	**Calculation of utilities:**	**Calculation of utilities:**	**Calculation of utilities:**
	-Utilities for EO and LO SMA models were derived from mapping PedsQL data from LO SMA patients enrolled in the CHERISH (SMA II) trial onto the EQ-5D scale	-Utilities in the LO model were derived from mapping PedsQL onto the EQ-5D scale using a published algorithm	Utilities in the LO model were derived from mapping PedsQL from SMA patients enrolled in the CHERISH (SMA II) trial on to the EQ-5D scale on to the EQ-5D scale using a mapping algorithm. For Biogen, these utilities lacked face validity and were not used for basic scenario analysis	-Utilities in the LO model were derived from mapping PedsQL from SMA patients enrolled in the CHERISH (SMA II) trial on to the EQ-5D scale on to the EQ-5D scale using a published algorithm
		-Values for infantile-onset model were based on later-onset utilities		-Values for infantile-onset model were based on later-onset utilities
Comments of the HTA body	The difficulty in obtaining QoL data from the early-onset patient population was acknowledged	—	-SMA II: CHERISH PedsQL data provides the most reasonable QoL measure	-Issues with methods calculating utilities: 1) Mapping of PedsQL scores to EQ-5D scores is a less valid method than measuring EQ-5D-3L scores directly, SF-6D, HUI, or domain or disease-specific questionnaires
			-SMA I: a reasonable estimate of QoL is to use adapted QoL CHERISH data	2) Mapping method has not been validated for this specific patient population
			-In it is reassessment TLV presented a range of utility values by considering utility values from the CHERISH trial on one end and utilities from an ALS study by Jones et al. (2014) on the other	3) PedsQL was used even for patients reaching adulthood
				-Scenario analyses explored different methods to determine utilities, rendering divergent outcomes. This generated uncertainty about QoL in the models
				-Rather optimistic estimation of long-term outcomes
Discounting	5% for costs and health outcomes. In the sensitivity analysis, the discount rate on costs and outcomes was set to 0 and 10%	—	3% for costs and health outcomes	4% for costs, 1.5% for outcomes
Comments of the HTA body	-Subgroup analysis indicated that cost-effectiveness is improved when nusinersen treatment was started at a disease duration and age of symptom onset of less than 12 weeks	-Assumptions for base case analysis maintain favorable outcomes for nusinersen (transition periods are maintained rather than applying a natural history rate, so no disease progression)	—	—
		-Overestimation of survival and QALY gains with nusinersen since transition probabilities are maintained indefinitely for nusinersen after the end of the trial, health states of patients on supportive care are expected to worsen		
		-Lack of long-term survival data		
		-ICER lies above the conventional ICER threshold, even considering economic evaluation weaknesses		

**Jurisdiction**	**England/Wales (NICE, 2018; NICE, 2019)**	**France (Haute Autorité de Santé, 2017)**	**The US (Ellis, 2019)**	**Canada (CADT common drug review, 2018)**
Perspective	Healthcare payer perspective	Healthcare payer perspective (health insurance and out-of-pocket expenses)	-Healthcare payer perspective	Healthcare payer perspective
			-Modified societal perspective as scenario analysis	
Economic evaluation technique	Cost-effectiveness analysis (LYG) + cost-utility analysis (QALY)	Cost-effectiveness analysis for EO (type I) SMA (LYG) + cost-utility analysis for LO (type II) SMA (QALY)	Cost-effectiveness (LYG) + cost-utility analysis (QALY)	Cost-utility analysis (QALY)
Comments of the HTA body	—	-Cost-utility analysis not reliable for SMA I due to insufficient information on QoL	Nusinersen was discussed in context of Zolgensma reimbursement	—
		-Cost-effectiveness analysis not relevant for SMA II in terms of LYG as impact on life expectancy is not demonstrated		
Economic model type (*de novo* Markov model structure)	2 models:	2 models:	3 models: -EO SMA (type I)	3 models: -EO SMA type I
	-EO SMA (type I)	-EO SMA (type I)	-LO SMA (type II and III)	-LO SMA type II
	-LO SMA (types II and III)	-LO SMA (only type II)	-Presymptomatic SMA	-LO SMA type III
Comments of the HTA body	-Positive outcomes for SMA types I, II, and III	-Not enough relevant information provided to perform cost-utility analysis of type III SMA patients	—	—
	-Biogen anticipates nusinersen to be the first-line treatment for all SMA patients despite lack of evidence on type 0 and IV SMA. Moreover, ERG’s clinical advisors stated that they would not treat these patients as they believe it unlikely that these patients would benefit from treatment			
Comparator (real-world care)	Standard of care, consisting of	Standard of care, consisting of	Standard of care, consisting of	Standard of care, consisting of
	-Respiratory care	-Respiratory care	-Respiratory care	-Respiratory care
	-Gastrointestinal care	-Gastrointestinal care	-Gastrointestinal care	-Nutritional care
	-Nutritional care	-Nutritional care	-Nutritional care	-Orthopedic care
	-Orthopedic care	-Orthopedic care		
		-Neurological care		
		-Palliative care		
Comments of the HTA body	-No data collection for patients treated with best supportive care, considered to be a significant limitation	-Choice of comparator is consistent with available data	—	—
	-In Biogen’s health economic analysis, the comparator is real-world care, which includes life-extending symptomatic care such as permanent respiratory support, whereas the comparator in the RCTs was a sham procedure (administered by lumbar puncture prick) in addition to best supportive care. Biogen stated that, for this reason, real-world survival may not reflect that seen in clinical trials. NICE prefers best supportive care (sham procedure and best supportive care)			
Time horizon	Lifetime horizon	-EO SMA (type I): 5 years	Lifetime horizon for both models (no further details)	-EO SMA type I: 25 years
	-EO SMA (type I): 40 years adjusted to 60 years (mean initial age: 5.58 months) as the company initially intended to use a 60-year time horizon	-LO SMA (only type II): 60 years	-EO SMA (type I) (mean initial age: 4.4 months)	-LO SMA type II: 50 years
	-LO SMA (types II and III): 80 years (mean initial age: 43.71 months)		-LO SMA (type II and III) (mean initial age: 2 years)	-LO SMA type III: 80 years
			-Presymptomatic (mean initial age: 21 days)	
Comments of the HTA body	—	Short time horizon for EO SMA patients seems conservative but is adapted to France where assisted ventilation is not used to prolong life of these patients	—	-Model does not adequately consider the impact of stopping nusinersen due to worsening of disease
				-The used time horizon was not appropriate for the scenarios considering Biogen reports specific ages
Target population	-EO (type I) SMA patients	-EO (type I) SMA patient subgroup: aged ≤7 months at inclusion and onset of symptoms ≤6 months after birth	-EO (type I) SMA patients	-EO (type I) SMA patients
	-LO (type II/III) SMA patients	-LO (type II) SMA patient subgroup: aged 2–12 years and onset of symptoms >6 months	-LO (type II and III) SMA patients	-LO (types II and III) SMA patients
			-Presymptomatic SMA patients	
Comments of the HTA body	-No evidence related to type 0 and IV SMA patients	-Distinction between type I and II patients acceptable but may be more complex in real life. The classification depends on the age of diagnosis and motorical capacities that can be obtained, yet in rare diseases, this may be compromised due to diagnostic delays that are observed in practice	-Nusinersen indicated for all SMA types despite only being studied in SMA types I, II, and III	-Clinical trial data are considered insufficient to support economic evaluation. Trial patients represent only a subset of SMA. There is especially a lack of data appropriate to assess effectiveness of nusinersen in SMA type III. Also, patient age in clinical trial (CT) more likely to favor response compared to real-world practice
		-Transposability of results to French practice is unknown	-Uncertainty regarding transferability of results from CT, with small patient samples and limited requirements for participation, to larger patient group	
			-Insufficient evidence for long-term safety and efficacy	
Scope of costs	-Direct medical costs: technology and health state maintenance, one-time end-of-life cost of 11,839 for EO (type I) SMA (informed by NICE guideline 61)	-Direct medical costs: technology and health state maintenance, end-of-life costs for EO (type I) SMA	**-Healthcare payer perspective:**	—
	-Direct nonmedical costs: transport and informal car	-Direct nonmedical costs: transport	-Direct medical costs: technology and health state maintenance	
			**Modified societal perspective:**	
			-Direct nonmedical costs (such as moving or modifying the home and purchasing or modifying a vehicle) and productivity gains for patients	
Comments of the HTA body	-Inclusion of end-of-life cost for late-onset patients	-Potential underestimation of administration costs	—	-Lack of transparency on reporting of cost estimates. Healthcare costs seem to be obtained from a German study by Klug et al. (2016)
				-Methods for extrapolation of costs of care to the Canadian context are limited. However, limited impact of additional healthcare cost expected given the costs of nusinersen
Outcomes	LYG and QALY gains, incl. patient and caregiver QALYs	SMA I: LYG (SMA I) and QALY gains (SMA II)	-Nusinersen was discussed in context of Zolgensma reimbursement	LYG and QALY gains
	**Calculation of utilities:**	Base case results including the caregiver perspective were not generated		**Calculation of utilities:**
	-PedsQL data from the CHERISH (SMA II) trial were converted to EQ-5D using a mapping algorithm by Khan et al.	**Calculation of utilities:**		-For SMA types I and III, a vignette study was used. The authors consulted SMA experts to provide health state descriptions. After, EQ-5D-Y was used by the experts to rate those health states made by the authors
	-The resulting utility values were adapted for the EO model based on an assumed correspondence of health states	-Utilities in the LO were derived from mapping PedsQL onto the EQ-5D scale using a published algorithm		-For SMA type II the utilities were derived from mapping PedsQL onto the EQ-5D scale using a published algorithm
		-Values for EO model were based on LO utilities		
Comments of the HTA body	-From available utility sources, the vignette study was preferred	-Limited transferability of early-onset CT data to current clinical French practice	—	-Utility values were, among others, derived from an unpublished study on QoL (Bastida et al.)
	-Company’s utility values had poor face validity (for instance, high valuations in poor health states)	-Calculated utility values were specific to the British population. Methodology used to obtain QoL data was not validated for a French population. Consistency between interpretation of British and French interpretation of QoL scores was questioned		-Inadequate methodology to estimate utility values:
	-The mapping algorithm used for PedsQL was limited (based on healthy children between 11 and 15 end very few responses for poor health states)			1) Experts should have established the health states instead of the authors. Experts can make different interpretations about the health states since “might have” is frequently used. 2) Mapping should be avoided and direct measurements are preferred according to the recent CADTH guidelines
	-Alternative utility values that were generated through a vignette study (Bastida et al.) do not have these methodological limitations but also had limited face validity (vignette study based on EQ-5D assessment by clinicians)			-Inappropriate assumptions relating to mortality for SMA I and II
	-Approach to generate caregiver disutilities not sufficiently justified and their inclusion introduces more uncertainty (unclear if health state impact is same for patient and caregiver, lack of face validity in patient utilities affects caregiver disutilities, calculations are arbitrary and based on other health states to the one being valued)			-Model includes relative states (related to the baseline patient characteristics) over absolute states such as the Hammersmith Functional Motor Scale-Expanded (HFMSE) scores, as preferred by the HTA body
Discounting	3.5% for costs and health outcomes	4% for costs and health outcomes	3% for the costs and health outcomes	1.5% for costs and health outcomes
Comments of the HTA body	—	-Potential underestimation of administration costs	-Uncertainty about long-term effects of repeated lumbar puncture	-Not enough data to conduct stratification by disease status within SMA type, for instance, stratified cost-effectiveness analysis by age
		-Uncertainty about long-term effects	-Trials of Spinraza® and Zolgensma® cannot be compared because the baseline properties differ	-Assumptions on long-term outcomes (disease progression, mortality) with nusinersen considered too optimistic:
		-Overall survival too optimistic	Medical chief of Cure SMA added additional comments:	1) All patients on nusinersen are assumed to improve even after the CT period whereas patients from the control group worsen/remain at the same level. Such assumptions regarding the disease progression are uncertain
		-Assumptions of “no deterioration” for nusinersen-treated patients and “no improvement” for patients treated according to the standard of care are too optimistic and do not reflect CT data	-Assumptions for the survival rates for the nonsitting group were incorrect	2) The assumption that 50% of the type III patients who cannot walk and are receiving nusinersen will gain ambulation after 24 months of CT period is not justified. CS2 and CS12 studies are not comparable whereby the effectiveness could not be proven in type III patients
			-Overall, ICER threshold is too low to meet challenges related to the rare disease (should be $500 000 instead of $150 000 per QALY)	3) Achieving milestones assumed to lead to reduced mortality, while there is no data proving this
			-The mentioned “controversies” in the CTs are common	

**TABLE 3:** Overview of the results of the economic evaluation of nusinersen in six European countries, the US, and Canada^a^.

Jurisdiction	Ireland (National Centre for Pharmacoeconomics (NCPE), 2017)		Scotland (SMC, 2018)		Sweden (NICE, 2018)		The Netherlands (Zorginstituut Nederland, 2018; Nederland Zorginstituut, 2019; Bruins, 2019)	
Institution	Biogen	National Centre for Pharmacoeconomics (NCPE)	Biogen	Scottish Medicines Consortium (SMC)	Biogen	Swedish Dental and Pharmaceutical Benefits Agency (TLV)	Biogen	Zorginstituut Nederland (ZIN)
Year of publication	2017	—	2017	—	2016	2016	2017	2017
**Base case analysis**								
Early-onset SMA								
Incremental LYG	—	—	5.55	—	—	2.11	7.28	—
Incremental QALYs	—	—	5.02	—	—	Between 1.78 and 1.33	5.93	—
ICER (€/LYG)	€463,726.07	—	—	—	—	€489,978.31	€431,214.38	—
ICER (€/QALY)	€512,843.80	—	€508,537.18	—	—	Between €583,035.82 and €779,435.06	€529,749.01	€632,801.85
Average incremental cost/patient	—	—	€2,550,615.71	—	—	—	€3,139,082.14	—
Early-onset SMA + caregiver utilities								
Incremental LYG	—	—	—	—	4.53	—	—	—
Incremental QALYs	—	—	—	—	Patient: 5.93; caregiver: 3.83	—	—	—
inflated ICER (€/LYG)	—	—	—	—	€467,850.36	—	—	—
ICER (€/QALY)	€253,502.37	—	€503,247.48	—	€217,142.00	—	—	—
Average incremental cost/patient	—	—	—	—	—	—	—	—
Late-onset SMA	—	—	—	—	—	Only SMA II	—	—
Incremental LYG	—	—	1.38	—	—	1.91	2.1	—
Incremental QALY	—	—	2.29	—	—	CHERISH: 3.02; Jones et al.: 5.33	3.53	—
ICER (€/LYG)	€3,998,625.72	—	—	—	—	€2.053.405,30	€1,873,658.78	—
ICER (€/QALY)	€2,156,623.69	—	€1,926,380.77	—	—	Between €736,298.03 and €1,297,144.31	€1,117,178.97	€1,792,938.58
Average incremental cost/patient	—	—	€4,419,838.74	—	—	—	€3,943,61.92	—
Late-onset SMA + caregiver utilities								
Incremental LYG	—	—	—	—	SMA II: 1.91; SMA III: 0	—	—	—
Incremental QALY	—	—	—	—	SMA II: 10.25 (patient); 1.61 (caregiver); SMA III: 1.63 (patient); 0 (caregiver)	—	—	—
ICER (€/LYG)	—	—	—	—	SMA II: €2,005,794.20	—	—	—
ICER (€/QALY)	€1,061,371.91	—	€1,365,539.29	—	SMA II: €322,857.74; SMA III: €1,564,889.39	—	—	—
Average incremental cost/patient	—	—	—	—	—	—	—	—
**Deterministic sensitivity analysis**								
Early-onset SMA (three most influential variables)	No tornado diagram provided, ICER is sensitive to	No tornado diagram provided, ICER is sensitive to	No tornado diagram provided	ICER is sensitive to mortality risk factors applied	No tornado diagram provided, ICER is sensitive to	No tornado diagram provided, ICER is sensitive to	Tornado diagram provided	—
	-Discounting %	Cost-effectiveness improvement when treatment given at			-Utility estimates	-Caregiver utility estimates	-Discounting % (costs and outcomes)	
	-Mortality risk factor	-Age at symptom onset <12 weeks				-Extrapolation of survival	-Vial price	
	-Vial price	-Disease duration<12 weeks				-Utility estimates	Month after patients still on treatment in “stands with assistance” stop improving	
	-Patient utility							
Late-onset SMA (three most influential variables)	No tornado diagram provided, ICER is sensitive to	—	No tornado diagram provided	ICER is sensitive to mortality risk factors applied	No tornado diagram provided, ICER is sensitive to	No tornado diagram provided, ICER is sensitive to	Tornado diagram provided	—
	-Discounting %				-Utility estimates	-Treatment interruptions	-Discounting % (costs and outcomes)	
	-Patient utility					-Time horizon	-Vial price	
	-Vial price					-Utility estimates	-Month after patients in the “stands/walks with assistance” stage stop improving or reach a plateau	
**Probabilistic sensitivity analysis**								
Early-onset SMA	Mean ICER: €498,480	—	—	—	—	—	Mean ICER (1,000 simulations): €503,740/QALY	—
							Cost-effectiveness probability (WTP of €80,000/QALY): 0%	
Late-onset SMA	Mean ICER: €2,107,108	—	—	—	—	—	Mean ICER (for 1,000 simulations): €1,082,249/QALY	—
	Including caregiver QALYs: €1,037,003						Cost-effectiveness probability (WTP of €80,000/QALY): 0%	
Comments of the HTA body	—	-Uncertainty on long-term treatment effectiveness efficacy	—	-Optimistic assumptions of the company regarding long-term treatment efficacy	—	Limited documentation available on:	—	-Large uncertainty regarding calculated ICERs due to long-term effects of nusinersen, utilities, and cost estimations; -Models estimate cost-effectiveness of SMA I, II, and III subgroups with relative short disease duration (this target population accords with optimized population scenario used for budget impact analysis). Therefore highly likely that ICER calculated by Biogen is the most optimistic/favorable scenario is (the lower limit ICER)
		-Uncertain translation of motor milestone gains to QALY gains		-Limited CT data regarding long-term survival		-Long-term effectiveness data		
		-Uncertain HRQoL assessment		-Uncertain modeling of long-term survival		-Swedish SMA population data		
		-Uncertain utility estimates (especially for SMA type I patients)				-QoL estimates		
						-Treatment continuation patterns.-SMA III patient’s population		

**Jurisdiction**	**England/Wales (NICE, 2018; NICE, 2019)**		**France (Haute Autorité de Santé, 2017)**		**The US (Ellis, 2019)**		**Canada (CADT common drug review, 2018)**	
Institution	Biogen	National Institute for Health and Care Excellence (NICE)	Biogen	The Economic and Public Health Committee (CEESP)	Biogen	Institute for Clinical and Economic Review	Biogen	Canadian Agency for Drugs and Technologies in Health (CADTH)
Year of publication	2016	2018	2017	—	—	2017	2017	2017
**Base case analysis**								
Early-onset SMA								
Incremental LYG	5.95	—	0.91	—	—	5.24	4.791	1.48
Incremental QALY	5.37	5.2	—	—	—	2.78	4.801	0.25
ICER (€/LYG)	—	—	€950,380.19	—	—	€550,343.00	—	—
ICER (€/QALY)	€492,349.77	€508,895.71	—	—	—	€1,037,256.64	€464,890.59	€6,399,097.41
Average incremental cost/patient	€2,642,072.75	—	€863,275.86	—	—	—	—	—
Early-onset SMA + caregiver utilities								
Incremental LYG	5.95	—	—	—	—	5.24	—	—
Incremental QALYs	5.44	3.47	—	—	—	2.78	—	—
ICER (€/LYG)	—	—	—	—	—	€400,164.66 (healthcare sector perspective)	—	—
						€555,939.71 (modified societal perspective)		
ICER (€/QALY)	€486,015.49	762,894.82	—	—	—	€755,555.65 (healthcare sector perspective)	—	—
						€1,048,450.06 (modified societal perspective)		
Average incremental cost/patient	€2,642,072.75	—	—	—	—	—	—	—
Late-onset SMA								
Incremental LYG	1.38	—	—	—	—	0	SMA II: 2.179	SMA II: 0
							SMA III: 0	SMA III: 0
Incremental QALYs	2.37	7.37	0.72	—	—	0.94	SMA II: 3.675	SMA II: 0.28
							SMA III: 1.563	SMA III: 0.56
ICER (€/LYG)	—	—	—	—	—	Spinraza dominated by BSC	—	—
ICER (€/QALY)	€1,513,499.17	€493,755,77	€2,719,821.37	—	—	€7,607,792.44	SMA II: €2,153,469.92	SMA II: €17,034,245.87
							SMA III: €1,994,745.73	SMA III: €4,189,626.81
Average incremental cost/patient	€3,580,776.32	—	€1,946,023.74	—	—	—	—	—
Late-onset SMA + caregiver utilities								
Incremental LYG	1.38	—	—	—	—	0	—	—
Incremental QALY	3.3	4.76	—	—	—	0.94	—	—
ICER (€/LYG)	—	—	—	—	—	Spinraza dominated by BSC	—	—
ICER (€/QALY)	€1,084,900.42	€764,425.24	—	—	—	€7,607,792.44	—	—
Average incremental cost/patient	€3,580,776.32	—	—	—	—	—	—	—
Presymptomatic SMA								
Incremental LYG	—	—	—	—	—	17.07	—	—
Incremental QALYs	—	—	—	—	—	15.69	—	—
ICER (€/LYG)	—	—	—	—	—	€608,175.66 (healthcare sector perspective)	—	—
						€589,519.96 (modified societal perspective)		
ICER (€/QALY)	—	—	—	—	—	€661,344.39 (healthcare sector perspective)	—	—
						€640,823.12 (modified societal perspective)		
**Deterministic sensitivity analysis**								
Early-onset SMA (three most influential variables)	Tornado diagram provided	No tornado diagram provided, ICER is sensitive to	Tornado diagram provided	—	—	No tornado diagram provided, ICER is sensitive to the utility when in the “sitting” health state and the healthcare costs in the “not sitting” health state	No tornado diagram provided	No tornado diagram provided
	-Vial price	-Utility estimates	-Nusinersen vial price					
	-Utility estimates (stands/walks unaided)	-Overall survival beyond CT time horizon	-Estimated hospitalization ratios					
	-Mortality risk factor	-Mortality rates applied	-Costs for neurologic and other care for type I					
Late-onset SMA (three most influential variables)	Tornado diagram provided	No tornado diagram provided, ICER is sensitive to	Tornado diagram provided	—	—	No tornado diagram provided	No tornado diagram provided	No tornado diagram provided
	-Utility estimate (“walks unaided” and “sits without support but does not roll”)	-Utility estimates	-Nusinersen vial price					
	-Vial price	-Mortality rates applied	-Utility estimate for patient state: worsened					
	-Mortality risk factor		-Utility estimate for walks unaided					
Presymptomatic SMA	—	—	—	—	—	No tornado diagram provided	—	—
**Probabilistic sensitivity analysis**								
Early-onset SMA	Mean incremental LYG: 5.9	Mean incremental QALY: 5.29	Mean ICER (cost/LYG): €937,209	—	—	—	For all three SMA types, the probability that nusinersen was cost-effective assuming an ICER threshold of $300,000/QALY was 0%	For all three SMA types, the probability that nusinersen was cost-effective assuming an ICER threshold of $300,000/QALY was 0%
	Mean incremental QALY: 5.32	Mean ICER (cost/QALY): £408,712; £404,270 (incl. caregiver QALY)	The probability that nusinersen was cost-effective was 80% for a willingness to pay of €1.25 million/LYG					
	Mean ICER (cost/QALY): £405,792	Mean incremental cost/patient: £2,160,048						
	Mean incremental cost/patient: £2,157,262	The probability that nusinersen was cost-effective at an ICER threshold of £337,000/QALY was approximately zero						
Late-onset SMA	Mean incremental LYG: 1.32	Incremental QALY: 2.28	ICER (cost/QALY): €2,570,106	—	—	—	The probability that nusinersen was cost-effective at an ICER threshold of $300,000/QALY was 0%	For all three SMA types, the probability that nusinersen was cost-effective at an ICER threshold of $300,000/QALY remained 0%
	Mean incremental QALY: 2.28	ICER (cost/QALY): £1,286,149; £933,088 (incl. caregiver QALY)	The probability that nusinersen was cost-effective is 80% for a willingness to pay of €3.13 million/QALY					
	Mean ICER (cost/QALY): £1,284,614	Incremental cost/patient: £2,938,441						
	Mean incremental cost/patient: £2,930,226	The probability that nusinersen was cost-effective at an ICER threshold of £500,000/QALY was approximately zero						
Comments of the HTA body	—	-No evidence relating to type 0 and type IV	—	SMA I: -Lack of QoL data available to conduct the recommended cost-utility analysis	—	—	—	-Findings of CDR reanalysis were similar to the manufacturer’s, in that nusinersen was not cost-effective for the three SMA types
		-Company’s implemented models are unnecessarily complex		-Limited transposability of clinical effectiveness results from England to France				-CDR reanalysis noted much higher ICURs. Results for type III SMA should be considered speculative given the lack of appropriate clinical data
		-Assumptions of no deterioration for nusinersen and no improvement for usual care are highly optimistic and do not reflect the observed trial data		-Unrealistic calculation of transition probabilities				
		-Assumptions regarding expected trajectory of nusinersen-treated patients through modeled motor milestone health states are highly favorable		SMA II: Unrealistic calculation of transition probabilities				
		-Assumptions on expected survival of patients on nusinersen are too optimistic. Company’s approach is complex, resulting in less transparency. Uncertainty regarding transferability of the results seen in the data sources as external data is used		-Great uncertainty associated with the reliability of QoL data collected				

a All costs were adjusted to 2019.

**TABLE 4:** Overview of budget impact analysis of nusinersen in five European countries and the US.

Jurisdiction	Ireland (National Centre for Pharmacoeconomics (NCPE), 2017)	Scotland (SMC, 2018)	Sweden (NICE, 2018)	The Netherlands (Zorginstituut Nederland, 2018, Nederland Zorginstituut, 2019; Bruins, 2019)	Belgium (RIZIV Dienst voor de Geneeskundige Verzorging; RIZIV Dienst voor de Geneeskundige Verzorging; RIZIV Dienst voor de Geneeskundige Verzorging; RIZIV Dienst voor de Geneeskundige Verzorging, 2018; Beleidscel van de minister van Sociale Zaken en Volksgezondheid, 2018)
Time horizon	5 years	—	—	3 years	3 years
Target population	—	-EO SMA: 5 patients in year 1, rising to 6 patients in year 5	Number of patients: 200–300	3 scenarios analyses	-SMA I: 18 patients in 2018 (incidence: 7 patients/year)
			-EO SMA type I: 6–9 patients	-Therapeutic added value scenario: 104 patients are qualified for treatment with nusinersen in 2020	-SMA II: 27 patients in 2017 (incidence: 3 patients/year)
		-LO SMA: 43 patients in year 1, rising to 48 patients in year 5	-LO SMA type II: 50–75 patients	-Optimized population scenario (if nusinersen is only available for those patients for who the clinical effect is highest)	-SMA III: 3 patients (incidence: 1 patient/year)
			-LO SMA type III: 200–250 patients	-Maximum scenario (nusinersen available for all patients)	
			No public data on the number of patients eligible for treatment with nusinersen		
Costs/patient	—	—	**Total costs per patient**	**Total costs per patient per year**	—
			-Cost/year per patient in year 1: €467,973.00	- €499,800.00/patient in year 1 for SMA I, II, or III	
			-Cost/year per patient in the following years: €233,987.00	- €249,900.00/patient in the following years for SMA I, II, or III	
Budget impact (BI) results	**Gross BI**	The BI analysis was conducted according to an MEA, more specifically a patient access scheme, negotiated with the company	After MEA proposal: no data on budget impact analysis publicly available	**From perspective 1: nusinersen alone (excl. standard of care)**	**Total BI for SMA type I, II, III, and presymptomatic patients (company estimates based on study population)**
	-EO SMA: €19,570,000.00			-BI in 2020: €29,738,100.00 for therapeutic added value, €23,240,700.00 for optimized scenario, €79,468,200.00 for maximum scenario	-BI year I: €40,000,000.00
	-LO SMA: €18,610,000.00 total gross: €38,180,000.00			**Broader perspective: nusinersen alone + drug administration costs (epidural injection)**	-BI year II: €25,000,000.00
	**Net BI**			-BI in 2020: €30,084,334.00 for therapeutic added value, €23,521,374.00 for optimized scenario, €80,163,523.00 for maximum scenario	-BI year III: €28,000,000.00
	-EO: €19,890,000.00			ZIN preferred scenario: patients for which nusinersen demonstrated added value (1,040 patients in 2020)	
	-LO: €17,990,000.00				
	**Total net BI:** €37,880,000.00				
Scope of costs	Report mentions “budget impact for nusinersen” without further specification	—	—	Perspective 1: budget impact for nusinersen, broader perspective: nusinersen and administration costs	—
Source of data	—	—	—	—	—
Comments of the HTA body	—	—	-Uncertain number of patients to be treated in the long-term. Within the target population, it is difficult to estimate those for which nusinersen might be relevant. Depends on, for instance, scoliosis surgery and mental state	-Uncertain number of eligible patients	-Uncertain percentage of patients who will stop treatment after 14 months
			-Uncertain treatment duration in clinical practice	-High additional costs when nusinersen added to package (29,700,000.00 in 2020)	-BI costs expected to be larger in real practice (type I: 85%, type II: 60%, and type III: 10%)
			-Also, patient numbers are expected to increase if nusinersen prolongs life of patients with severe SMA	-Lifelong treatment needed	
				-Recommended pay-for-performance agreement	

**Jurisdiction**	**Germany (IQWiG, 2017)**	**France (Haute Autorité de Santé, 2017)**	**The US (Ellis, 2019)**	**Canada (CADT common drug review, 2018)**	
Time horizon	—	5 years (2017–2021)	5 years (2019–2023)	—	
Target population	Number of patients (type I-IV SMA): 841–1,061	Number of patients	-EO SMA type I: 215 new patients each year of which 75% eligible for treatment with Spinraza vs. 25% with BSC in absence of Zolgensma	—	
	-EO SMA type I: 70–120 patients	-SMA I: 114 patients			
	-LO SMA type II: 360–440 patients	-SMA II: 392 patients			
	-LO SMA types III-IV: 410–500 patients	-SMA III: 454 patients			
		Estimated number of new patients/year: 80–130			
		SMA type IV patients are excluded from the analyses as these patients are not expected to be treated with nusinersen, although eligible to nusinersen treatment according to MA indication			
Costs/patient	**Total costs per patient**	—	**Total costs per patient**	**Drug costs (presumably per patient)**	
	- €621,354.48/year 1		-Presymptomatic SMA: $573,900.00 per year	-$708,000.00/year 1	
	- €310,877.58€–€310,942.95/subsequent year			-$354,000.00/subsequent year	
Scope of data	—	—	—	—	
Comments of the HTA body	The annual therapy costs for maintenance therapy correspond to the costs of medicines and the costs of additionally required health insurance services (lumbar puncture)	-Health insurance perspective for type I, II, and III SMA patients (different perspective compared to the economic evaluation)	—	-Drug costs (no further info)	
Source of data	—	—	—	—	
Comments of the HTA body	-Uncertain number of patients in the GKV target population, although largely plausible	-Underestimation of nusinersen doses administered	—	—	
	-Uncertainty regarding transferability of the prevalence rate for EO SMA from 1987 to the current care as well as prevalence rate for LO SMA derived from Norwood et al. study	-Potential underestimation of eligible population due to intransparent and irreproducible calculation method. For instance, the estimation for EO SMA type I patients, eligible for treatment with nusinersen, excluded a proportion of patients expected to develop arthrodesis, which is a contra-indication for nusinersen. On the other hand, EO SMA type I patients have a life expectancy of below the age of 2 years while arthrodesis is expected to occur around the age of 12			
		-99% of BI costs associated with nusinersen acquisition (50% vial price reduction implies 50% decrease in BI)			

### Inflation and Currency Adjustment

In the base case analysis, all costs were adjusted for inflation and currency changes using the methodology described by Turner et al. (2019). We first inflated costs in the local currency, by using local inflation rates for 2019 for Canada, United Kingdom, Ireland, the US (News, 2019), and Sweden (Exchange Rates, 2021). For inflation, we used the Gross Domestic Product (GDP) implicit price deflators which are published annually by the World Bank (The World Bank Group, 2021). Then, local currency values were exchanged. The costs included in the probabilistic sensitivity and budget impact analysis were not adjusted for currency and inflation.

## Results

In the following sections, we have summarized, per jurisdiction, several aspects of the economic and budget impact analysis, together with information on the MEA and the reimbursement decision. This is followed by a comparative analysis of the economic evaluation, the budget impact analysis, and reimbursement decision over the different jurisdictions. The full details of the design of the economic analysis, its outcomes, and the budget impact analysis are presented in Tables 2–4, respectively.

### Ireland

The National Centre for Pharmacoeconomics (NCPE) based its assessment report on nusinersen on the economic evaluation as submitted by Biogen. The economic evaluation included two Markov models, 1) for early-onset (EO) (type I) and 2) late-onset (LO) (types II and III) SMA, comparing nusinersen to the standard of care. Life years gained (LYG), patient’s quality-adjusted life years (QALY), and caregiver QALYs were included for both models and calculated over a lifetime horizon. Direct medical costs (technology and health state maintenance) were included. Biogen obtained utilities for both EO and LO SMA by deriving Pediatric Quality of Life Inventory (PedsQL) data from LO SMA patients enrolled in the CHERISH (SMA II) trial. PedsQL is a questionnaire developed to measure the health-related QoL in children and adolescents (Varni et al., 1999). Later, these data were mapped onto the EQ-5D scale. Here, NCPE acknowledged the difficulty of obtaining utilities for EO SMA patients. Discount rates for costs and outcomes were set at 5%. The report presented the results of the base case, sensitivity, and scenario analyses. For the base case analysis, a healthcare payer perspective was adopted, whereas for the scenario analyses, a societal perspective was considered. ICERs were €512,844/QALY and €2,156,624/QALY for EO and LO SMA, respectively. With caregiver utilities included, values dropped to €253,502/QALY and €1,061,37/QALY, respectively. Subgroup analysis indicated that cost-effectiveness could be improved if nusinersen treatment was started when both disease duration and age of symptom onset were less than 12 weeks. Although the report does not present a tornado diagram, the sensitivity analysis indicated a great impact of the discount factor, the nusinersen vial price, and patient utilities for both EO and LO SMA and, for EO SMA additionally, the mortality risk factor. The probabilistic sensitivity analysis resulted in a mean ICER of €498,480 for EO SMA, €2,107,108 for LO SMA, and €1,037,003 for LO SMA with caregiver QALYs included.

Overall, the NCPE concluded nusinersen to be not cost-effective in either EO or LO SMA. They found that a 10- and 20-fold price reduction would be necessary for nusinersen to either approach the €45,000/QALY threshold for EO SMA or fall below the €100,000/QALY threshold for LO SMA, respectively. The total net budget impact for treatment with nusinersen was estimated at €37.88 million, being €19.89 million and €17.99 million for EO and LO SMA, respectively, although the report did not specify whether this includes administration and/or health maintenance costs. Ultimately, the NCPE did not recommend reimbursement of nusinersen at the submitted price, based on its cost-effectiveness and budget impact. Still, nusinersen was granted reimbursement for patients under 18 years old with SMA types I, II, and III after confidential price negotiations were finalized (Ryan, 2019a).

### Scotland

The assessment of the Scottish Medicines Consortium (SMC) was, in part, based on the economic evaluation results for which Biogen submitted two Markov models, for EO and LO SMA, comparing nusinersen to the standard of care. SMC highlighted Biogen’s exclusion of presymptomatic SMA patients from its application. Both models took a lifetime horizon, set at 40 and 80 years for EO and LO SMA, respectively. Biogen obtained LO SMA utilities by mapping PedsQL data, derived from LO SMA patients enrolled in the CHERISH (SMA II) trial, onto the EQ-5D scale. EO SMA utility values were based on those obtained for LO SMA, with minor adaptations as they were regarded as “sufficiently similar” to infants. Discount rates for costs and outcomes were not specified. The report presented results of the base case analysis only, adopting a healthcare payer perspective. ICERs were €508,537/QALY and €1,926,381/QALY for EO and LO SMA, respectively. Additionally, Biogen performed a number of scenario analyses, adopting a societal perspective which included caregiver utilities and costs. Compared to the base case, these ICER values dropped to €503,247/QALY and €1,365,539/QALY for EO and LO SMA, respectively. Scenario analysis highlighted the ICER’s sensitivity to the mortality risk factor that was adopted in both EO and LO SMA models, although the report did not present a tornado diagram or any other results. Overall, the SMC highlighted several key limitations of the economic evaluation, such as the lack of long-term survival data, optimistic assumptions of overall survival for patients receiving nusinersen and their utility values, especially given the fact that the model assumed that transition probabilities are maintained indefinitely for patients treated with nusinersen while those receiving the standard of care worsen over time.

In its advice, the SMC also considered the views of the Patient and Clinician Engagement (PACE) meeting, during which patients shared their experiences on the disease burden for both patients and caregivers and where they highlighted the impact of SMA on patient’s ability to live independently and develop a career.

Biogen proposed an MEA which was deemed acceptable for implementation in Scotland. The SMC added that it wished to report on the cost-effectiveness estimates as obtained under the MEA, yet was unable to do so due to confidentiality reasons. As the budget impact analysis was performed in the context of the MEA, no data were presented on budget impact other than the estimated real-life target population.

For all elements considered, the SMC argued that the ICER values for both EO and LO SMA exceeded conventional ICER thresholds while there was still economic uncertainty. However, nusinersen met certain requirements that acted as decision-modifying criteria, namely, the absence of alternative treatment and the substantial improvement of life expectancy in EO SMA. These disease-modifying criteria allowed the SMC to accept greater economic uncertainty associated with reimbursing nusinersen; thus, access to the treatment was granted for patients with EO SMA. Later, access was extended to patients with LO SMA (SMA News Today, 2019; TreatSMA, 2021).

### Sweden

The Swedish Dental and Pharmaceutical Benefits Agency’s (Tandvårds-och läkemedelsförmånsverket, TLV) advice on the reimbursement of nusinersen was partly based on the results of the economic evaluation, comparing nusinersen (and standard treatment) to the standard of care. Biogen submitted results for three Markov models, for EO type I, LO type II, and LO type III SMA. For EO (type I) and LO (type II and III) SMA, both LYG and patient and caregiver QALYs were calculated over a lifetime horizon, set at 40 and 80 years, respectively. Utilities were calculated by mapping PedsQL outcomes onto the EQ-5D scale by using a published algorithm. Both costs and outcomes were discounted at 3%. The base case analysis adopted a societal perspective, including direct medical and nonmedical costs. Additionally, caregiver productivity loss was included as an indirect cost. This resulted in ICERs of €17,142/QALY; €322,858/QALY; and €1,564,889/QALY for EO type I, LO type II, and LO type III, respectively. These ICERs were most sensitive to utility estimates, although a tornado diagram was not provided. TLV excluded caregiver utilities from its base case reanalysis, reporting ICER ranges from €583,035/QALY to €8,251,567/QALY for EO and €736,298/QALY to €1,297,144/QALY for LO type II SMA (depending on utility values used). TLV did not include LO type III SMA outcomes as these data were obtained from noncontrolled studies. ICERs calculated by TLV were higher as its model was based on different assumptions. It tested, for instance, more realistic assumptions regarding disease progression for nusinersen patients, such as a lower probability of death for patients on nusinersen compared to the standard of treatment. Overall, TLV noted uncertainties regarding long-term effectiveness, extrapolation of data, utility estimates and continuation of treatment.

Information on budget impact was limited to the cost/patient/year, amounting to €467,973 in year one and to €233,987 in subsequent years. The number of patients per SMA subgroup was reported although the number of patients eligible for nusinersen treatment was kept confidential. Here, TLV pointed out uncertainties regarding the number of patients eligible for treatment in the long term and the treatment duration. They noted that these numbers are expected to increase in the future after nusinersen prolongs the life of patients with severe SMA.

TLV considered the cost/QALY to be too high in order to provide access according to the European label. They therefore recommended providing access only to patients for whom studies have shown treatment benefit. Hence, access was granted to type I and II SMA patients under 18 years old, and to patients with a subtype of type III (type IIIa) SMA, who are younger than 3 years of age and have a disease pattern comparable to SMA type II (NT-council, 2019; Janusinfo, 2021).

### England and Wales

The National Institute for Health and Care Excellence (NICE) published its report on the single technology appraisal of nusinersen in 2018. In Wales, the All Wales Medicines Strategy Group (AWMSG) excluded nusinersen from assessment and adopted the reimbursement decision made by NICE (AWMSG, 2021). An economic evaluation was included in Biogen’s submission, presenting two Markov models, for EO (type I) and LO (types II and III) SMA, respectively. Incremental LYGs as well as both incremental patient and caregiver QALYs were calculated over a lifetime horizon. The models included direct medical (including a one-time end-of-life cost for SMA I) and nonmedical costs. Utilities in LO SMA were calculated by mapping PedsQL outcomes onto the EQ-5D scale by using a published algorithm. EO SMA utilities were derived from those for LO SMA and based on an assumed correspondence of health states between EO and LO SMA. Costs and outcomes were discounted at 3.5%. The base case analysis adopted a healthcare payer perspective, resulting in ICERs of €492,350/QALY and €1,513,499/QALY for EO and LO SMA, respectively. With caregiver utilities included, these values dropped to €486,015/QALY and €1,084,900/QALY, respectively. The tornado diagram showed that, for EO SMA, the factors that influenced the ICER most were the vial price, the utility estimates for the best and worst health states, and the mortality adjustment factor applied to better health states. The probabilistic sensitivity analysis resulted in a mean ICER of £405,792/QALY for EO SMA and £1,284,614 for LO SMA.

NICE noted the fact that there was no evidence submitted that was related to type 0 (*in utero* onset) and IV (adult onset) SMA. Biogen reportedly stated that SMA type 0 and IV patients were omitted from submission as the clinical evidence available at that time would not meet appraisal requirements. Still, they anticipated nusinersen to be reimbursed and available for first-line treatment of all SMA patients. However, NICE’s clinical advisors stated that they would not treat type 0 SMA patients, except in the context of clinical trials, nor that they would treat type IV SMA patients with nusinersen, as they found it unlikely for these patients to benefit from treatment. Furthermore, the PedsQL mapping algorithm was considered to be limited, for instance, because it was based on healthy school children between age 11 and 15 and because of the few responses of patients in poor health states. Alternative utility values, deducted from a vignette study, were available. Although these also had limited face validity, they did not have the same methodological limitations and thus were considered most appropriate. NICE also noted several shortcomings with the calculations of caregiver disutilities. They criticized Biogen’s assumptions on probabilities of transitioning from one health state to another and on overall survival. For instance, in Biogen’s model, nusinersen patients could not deteriorate, while patients treated with usual care could not improve. These assumptions are inconsistent with trial data that showed a portion of nusinersen patients transitioning to a worse health state, while a proportion of patients receiving usual care improved.

Reanalysis by NICE found ICERs that were higher for EO SMA, yet much lower (one-third) for LO SMA compared to those presented by Biogen. In both EO and LO SMA the inclusion of caregiver QALYs led to an increase of the ICER as calculated by NICE. Overall, ICERs were €508,896/QALY and €493,756/QALY for EO and LO SMA, respectively, and €762,895/QALY and €764,425/QALY with caregiver QALYs included. The presented ICERs were sensitive to utility estimates and mortality rates in both models and to the overall survival beyond the clinical trial’s time horizon for EO SMA, although the report did not present a tornado diagram. For EO SMA, the probabilistic sensitivity analysis resulted in a mean ICER of £408,712/QALY and £1,286,149 for EO and LO SMA, respectively. With caregiver QALYs included, these values dropped to £404,270/QALY and £933,088, respectively. The results showed a 0% chance for nusinersen to be cost-effective at a threshold of £337,000/QALY for EO and £500,000/QALY for LO SMA. The report did not include information on budget impact.

In order to address long-term uncertainties, Biogen proposed an MEA for a 5-year term and included eligibility and stopping criteria in its draft proposal. Data collection is proposed after 14 months initially and 12 months afterward. The outcomes calculated are survival, ventilation/respiratory events, motor function, and the QoL (for both patients and caregivers). They are collected through the SMART NET registry, including patients who discontinue nusinersen. However, NICE remarked on Biogen’s intention to not include comparative data on patients receiving standard of care, which was considered a significant limitation. They also mentioned the lack of outcome collection for patients with type 0 or IV SMA. NICE concluded that nusinersen was not cost-effective. An MEA was set up, consisting of a price discount combined with coverage with evidence development (CED) agreement (NICE, 2019). After the agreement was reached, NICE recommended nusinersen for reimbursement in presymptomatic and type I, II, and III SMA, for the duration of and within the conditions set out in the MEA (Coyle, 2021).

### France

In France, the value assessment of nusinersen was performed by the Economic and Public Health Evaluation Commission (Commission Evaluation Economique et de Santé Publique, CEESP), which issued an advice to the French National Authority for Health (Haute Autorité de santé, HAS) and finalized its report on December 12, 2017. The advice included the assessment of the economic evaluation and its results as submitted by Biogen. Two Markov models were presented, for EO (type I) and LO SMA (type II), of which the former calculated LYGs and the latter patient QALYs calculated over a time horizon of 5 and 60 years, respectively. Both direct medical and nonmedical costs were included. Utilities in LO SMA were calculated by mapping PedsQL outcomes onto the EQ-5D scale by using a published algorithm. EO SMA utilities were based on those from LO SMA. Both costs and outcomes were discounted at 4%. The base case analysis adopted a healthcare payer perspective, presenting ICER values of €950,380/QALY and €2,719,821/QALY for EO and LO SMA, respectively. The tornado diagram showed that the nusinersen vial price was the most influential factor for both EO and LO SMA, followed by the estimated hospitalization ratios (nusinersen versus real-world care) and costs for neurologic and other care for EO (type I) SMA and the utility estimate for several health states for LO (type II) SMA. The probabilistic sensitivity analysis resulted in a mean ICER of €937,209/LYG and €2,570,106/QALY for EO and LO SMA, respectively. Overall, the models showed an 80% probability for nusinersen to be cost-effective at a threshold of €1.25 million per LYG and €3.13 million per QALY for EO (type I) SMA and LO (type II) SMA, respectively.

CEESP noted limited transferability of EO (type I) SMA trial data to French current practice, as well as of utilities, which were calculated in the British population. Moreover, the method to obtain the utilities was not validated for the French population. The time horizon over which utilities were calculated in EO (type I) SMA was deemed conservative, although it may align to real healthcare practice in France where the life of EO SMA patients is not extended by using assisted ventilation. Overall, CEESP highlighted the lack of data to consider the long-term effects of nusinersen. They found that the estimation of QALYs in EO (type I) SMA was not relevant due to the lack of data on the QoL. Furthermore, they found that the impact of nusinersen on life expectancy in LO (type II) SMA was not demonstrated and therefore considered LYGs irrelevant for this SMA subgroup. Moreover, they stated that the assumptions related to overall survival were too optimistic, in particular the assumption that SMA type II patients on nusinersen will not deteriorate, while those receiving standard of care will not improve. They further noted that the costs for administrating nusinersen were potentially underestimated. In its report, CEESP did not provide a judgment on the cost-effectiveness of nusinersen.

The budget impact analysis adopted a health insurance perspective, and CEESP remarked a potential underestimation of both nusinersen doses administered as well as the total number of patients eligible for treatment. They further noted that 99% of all costs are associated with nusinersen acquisition. Final budget impact data were not presented. When the report was published, nusinersen had a temporary reimbursement status under the conditions of an ATU (temporary authorization of use) plan, which grants exceptional access to medicinal products before a centralized market authorization is granted by EMA (Launet et al., 2004). Ultimately, reimbursement was maintained for patients with EO type I and LO type II and III SMA, although no information is available on the reimbursement conditions that were agreed upon in the MEA.

### The United States

In the US, a report was issued by the Institute for Clinical and Economic Review on April 3, 2019 (updated on May 24), that evaluated nusinersen in the context of the reimbursement of onasemnogene abeparvovec (Zolgensma®). At that point, nusinersen was already reimbursed depending on the patient’s insurance provider (Biogen, 2019). The document reports on the results of the economic evaluation as presented by three Markov models, for EO (type I), LO (types II and III), and presymptomatic SMA, each comparing nusinersen to the standard of care. Each model included LYG and QALYs, calculated over a lifetime horizon for each SMA subtype, although no further details were provided. Utility values were derived from multiple sources. Costs and outcomes were discounted at 3%. Results of the base case analysis were presented, adopting a healthcare payer perspective, including direct costs. The report presented ICERs of €1,037,257/QALY; €7,607,792/QALY, and €608,176/QALY for EO, LO, and presymptomatic SMA, respectively.

With caregiver utilities included, these values decreased to €755,556/QALY for EO SMA, yet remained the same for LO SMA (€7,607,792/QALY). A number of scenario analyses were performed, one of which adopted a modified societal perspective, including nonmedical costs and productivity gains for patients. The economic evaluation from the healthcare payer perspective and from the modified societal perspective resulted in similar ICERs. The institute did not comment on the cost-effectiveness of nusinersen.

It was noted that, for EO SMA, the ICER was sensitive to the utility when in the “sitting” health state and to the healthcare costs in the “not sitting” health state, although the report did not present a tornado diagram. The Institute for Clinical and Economic Review commented on the lack of evidence on long-term safety and efficacy, for instance, on the long-term effects of repeated lumbar puncture for nusinersen administration. Furthermore, the institute questioned whether the small clinical trial patient group and limited requirements to participate in clinical trials allow generalizability of the results to a broader patient group. The report did not show budget impact data on nusinersen treatment compared to the standard of care.

### The Netherlands

Zorginstituut Netherland (ZIN) issued its final advice on the reimbursement of nusinersen on February 5, 2018. Its advice was based on several appraisal criteria and reported on efficacy, therapeutic need, cost-effectiveness, and budget impact. The data submitted by Biogen included an economic evaluation based on two Markov models, for EO (type I) and LO (type II and III) SMA, comparing nusinersen to the standard of care. Both models included incremental LYGs and patient QALYs, calculated over a lifetime horizon, set at 40 and 80 years for EO and LO SMA, respectively. The target population of the economic evaluation corresponded to the trial population (ENDEAR and CS3A), which represented a subgroup of both EO (type I) and LO (type II and III) SMA. The scope of costs included direct medical, as well as direct and indirect nonmedical costs. Utilities in LO SMA were calculated by mapping PedsQL outcomes onto the EQ-5D scale by using a published algorithm. EO SMA utilities were based on those from LO SMA. Costs and outcomes were discounted at 4 and 1.5%, respectively. Results of the base case analysis were presented, adopting a societal perspective, and reported ICER values of €529,749/QALY and €1,117,179/QALY for EO and LO SMA, respectively. Biogen found that these values were most influenced by the discount factor and vial price (for both EO and LO SMA), as well as the month after which a specific motor milestone was reached. Biogen’s probabilistic sensitivity analysis showed a mean ICER of €503,740/QALY and €1,082,249/QALY for EO and LO SMA, respectively. In addition, they estimated a 0% probability that nusinersen is cost-effective at an ICER threshold of €80,000 per QALY in both EO and LO SMA.

Analysis by ZIN concluded that the classification of the EO (type I) SMA subgroup as defined in the model’s target population corresponded to Dutch clinical practice, while the classification of the LO SMA subgroup (types II and III) was found to not correspond to type III patients in Dutch clinical practice. On the other hand, ZIN noted that the data submitted by Biogen represents, per SMA type (I, II, and II), only a subgroup of patients, who had a relatively short disease duration and thus started treatment earlier in comparison to real-world treatment. Therefore, Biogen’s ICER was assumed to reflect the lower limit ICER, hereby representing the most favorable scenario. Reanalysis by ZIN found higher ICER values than those presented by Biogen: €632,802/QALY and €1,792,939/QALY for EO and LO SMA, respectively. Also, ICERs were considered to be highly uncertain due to uncertainties on utilities, long-term outcomes, and (methods for) cost estimations of treatment with nusinersen. With respect to utilities, the applied mapping method was (at that time) not validated in the target population and Biogen was recommended to measure EQ-5D scores either directly or through disease-specific questionnaires. Biogen’s estimation of long-term outcomes was considered to be rather optimistic. ZIN questioned the appropriateness of the methodology used to calculate productivity loss. In addition, it found major differences in cost/year per SMA type between the studies that Biogen used as a source of cost data, which further raised uncertainties on the methods used to define these costs.

Budget impact data were presented for three scenarios, 1) the optimized population scenario (treatment for patients with highest clinical effects), 2) the therapeutic added value scenario (patients for which nusinersen demonstrated added value), and 3) the maximum scenario (treatment for all SMA patients). For each scenario, the budget impact was estimated for the cost of nusinersen alone (thus excluding standard of care) and for costs of nusinersen treatment including drug administration costs (consisting of a lumbar puncture). For the added value scenario, which was preferred by ZIN, the budget impact was estimated at €29.74 million for nusinersen alone and €30.08 million with administration costs included. ZIN remarked the high additional costs of adding nusinersen to the health insurance package, commenting on the uncertainty concerning the total number of eligible patients and the fact that they will need lifelong treatment with nusinersen. Therefore, a pay-for-performance (P4P) agreement was recommended, which provides reimbursement only when treatment is found effective (and thus according to the added value scenario).

Overall, ZIN found nusinersen to be not cost-effective and therefore did not recommend reimbursement unless price negotiations (a price decrease of at least 85%) led to an improvement of the cost-effectiveness. Nevertheless, both the high unmet need and the solidarity principle were arguments that played in favor of reimbursement. However, ZIN pointed out that reimbursement will ultimately threaten the solidarity principle, voicing concern that the combination of a high budget impact with uncertain cost-effectiveness risks displacing other care. Together with Belgium, a joint MEA was set up, linking reimbursement to nusinersen’s performance while reducing the cost through a price discount. Hence, nusinersen was made available for EO SMA type I, LO SMA types II and III, and presymptomatic patients, yet only when an added value in these patients is demonstrated. Meanwhile, the agreement required Biogen to collect additional data on the long-term effectiveness and safety in real-life practice. For patients with SMA who are older than 9.5 years, reimbursement was granted conditionally for 7 years, yet the price was kept confidential (Bruins, 2019).

### Canada

In January 2018, the Canadian Agency for Drugs and Technologies in Health (CADTH) issued its evaluation report on nusinersen. Biogen’s submission was based on three Markov models: one for EO type I, one for LO type II, and one for LO type III SMA, each comparing nusinersen to the standard of care. Each model included LYG and patient QALYs that were calculated over a lifetime horizon, set at 25, 50, and 80 years for EO type I, LO type II, and LO type III SMA, respectively. However, for each subtype, only the cost/QALY was presented. Biogen obtained utilities for LO type II SMA patients by mapping PedsQL data, derived from LO SMA patients enrolled in the CHERISH (SMA II) trial, onto the EQ-5D scale. For EO type I and LO type III SMA, Biogen estimated utilities using a vignette study, where authors asked five SMA experts to describe health states, which they then rated according to the EQ-5D questionnaires. Discount factors for costs and outcomes were set at 1.5%. The report presented the results of the base case analysis and probabilistic sensitivity analysis, adopting a healthcare payer perspective.

CADTH noted several shortcomings related to the methods applied to calculate utilities and concluded that they were deemed inappropriate, as direct measurements were preferred according to CADTH guidelines. Furthermore, CADTH found Biogen’s assumptions on long-term outcomes too optimistic for patients on nusinersen and noted that overall, the clinical trial data were insufficient to support the economic evaluation, since patients enrolled in the trial presented only a subset of SMA, for whom a more favorable response was more likely when compared to real-life treatment. They also highlighted a lack of data to estimate the cost-effectiveness in LO type III SMA or to conduct stratification by diseases status.

ICERs were €464,891/QALY; €2,153,470/QALY; and €1,994,746/QALY for EO type I, LO type II, and LO type III SMA, respectively. The report did not include a tornado diagram and did not mention the most influential variables affecting the ICER. The probabilistic sensitivity analysis showed 0% probability for nusinersen to be cost-effective at a $300,000/QALY threshold. The results of the CADTH reanalysis were in line with Biogen’s findings, more specifically, a 0% chance for the cost-effectiveness of nusinersen to be below the $300,000/QALY threshold. Moreover, CADTH found much higher ICERs (€6,399,097/QALY for EO type I SMA; €17,034,246/QALY; and €4,189,627/QALY for LO type II and III SMA, respectively), yet they provided no justification for this discrepancy. They did note that the results for LO type III SMA should be considered speculative due to the lack of appropriate clinical data.

No information on the budget impact analysis was presented other than the drug cost/patient, which was calculated at $708,000 and $354,000 for the first and for each subsequent year, respectively.

Overall, although CADTH concluded that nusinersen was not cost-effective, reimbursement was granted under certain conditions and dependent on the province.

### Belgium

The reimbursement reports of the National Institute for Health and Disability Insurance (RIZIV/INAMI) did not include any data on the cost-effectiveness of nusinersen, as reimbursement decisions for orphan drugs in Belgium do not require an economic evaluation. Budget impact data were included, presenting company estimates based on the study population, generating a total cost of nusinersen of €40 million in year 1, and lowering to €25 million and €28 million for the second and third year, respectively. RIZIV/INAMI commented that it expects these estimates to be larger in real-life practice. They noted, for instance, that the percentage of patients who will stop treatment after 14 months remains uncertain. Still, nusinersen was granted reimbursement for patients with type I, II, and III SMA, including presymptomatic patients. A combined financial/outcome-based MEA was set up, involving a price reduction and allowing access to patients for whom an added value was demonstrated. However, the total cost was managed through an absolute cap, which allowed managing uncertainties concerning the total number of eligible patients. Also, it was defined that RIZIV/INAMI would not reimburse costs for nonresponders or all extra costs made during the first year to initiate patients. Additionally, Biogen agreed to collect additional long-term effectiveness and safety data.

### Italy

The Italian Medicines Agency (Agenzia Italiana del Farmaco, AIFA) did not assess the cost-effectiveness or budget impact of nusinersen. Rather, reimbursement decisions of medicines in general are based on an assessment of therapeutic need, added therapeutic value and disease rarity. AIFA noted reservations regarding data quality. Based on the overall assessment, nusinersen was granted the status of “therapeutic innovation” and received reimbursement for patients with EO type I and LO type II and III SMA patients, hereby excluding patients with more than four copies of the SMN2 gene (AIFA, 2017a; AIFA, 2017b).

### Germany

In Germany, reimbursement status depends on a drug’s added benefit, which is *de facto* considered proven for those with an orphan designation. Additionally, this status is retained as long as the total turnover amounts to a maximum of €50 million within 12 calendar months. Hence, nusinersen was already reimbursed at the time of assessment. However, as the orphan drug’s turnover exceeded €50 million, the Institute for Quality and Efficiency in Health Care (Institut für Qualität und Wirtschaftlichkeit im Gesundheitswesen, IQWiG) did perform an assessment on the extent of the added benefit and issued its final advice to the Federal Joint Committee (Gemeinsamer Bundesausschuss, G-BA) (IQWiG, 2017; G-BA, 2021). IQWiG estimated the cost/patient at €621,354 for the first year and between €310,878 and €310,943 for each subsequent year. They concluded a significant and substantial benefit for EO type I and LO type II SMA, respectively. The added benefit for SMA patients with LO type III and IV was considered nonquantifiable due to the lack of QoL data in these SMA subtypes. Therefore, the final decision was based on other factors such as mortality, morbidity, and risk of adverse events. On these grounds, nusinersen remained reimbursed for the treatment of all SMA patients.

### Comparative Analysis

#### Economic Evaluation

The reimbursement reports for nusinersen in all jurisdictions, with the exception of Belgium, Italy, and Germany, included the results of the economic evaluation. Depending on the jurisdiction of submission, Biogen provided either two or three *de novo* Markov models, presenting results either for EO type I separately from those for LO type II and III SMA or separately for all three SMA subtypes. HTA bodies in both France and Sweden considered the data on QoL in LO SMA type III to be weak. Additionally, both Scotland and England and Wales reported the lack of data on type 0 and IV SMA. In the US, a third Markov model included presymptomatic SMA, although the report was not based on a file submitted by Biogen.

In each jurisdiction, Biogen calculated costs and outcomes over a lifetime horizon and this choice was considered to be appropriate by the respective HTA bodies. The target population presented by Biogen corresponded to the clinical trial population, where patients were excluded based on their age and other criteria such as co-occurring scoliosis or issues with mental health. Hence, the target population represented only a subgroup of SMA patients, for whom a favorable response with nusinersen treatment is more likely when compared to real-world practice. Furthermore, France, England and Wales, and Sweden questioned the extrapolation of data due to a lack of local data on costs and patient numbers.

**FIGURE 1:**
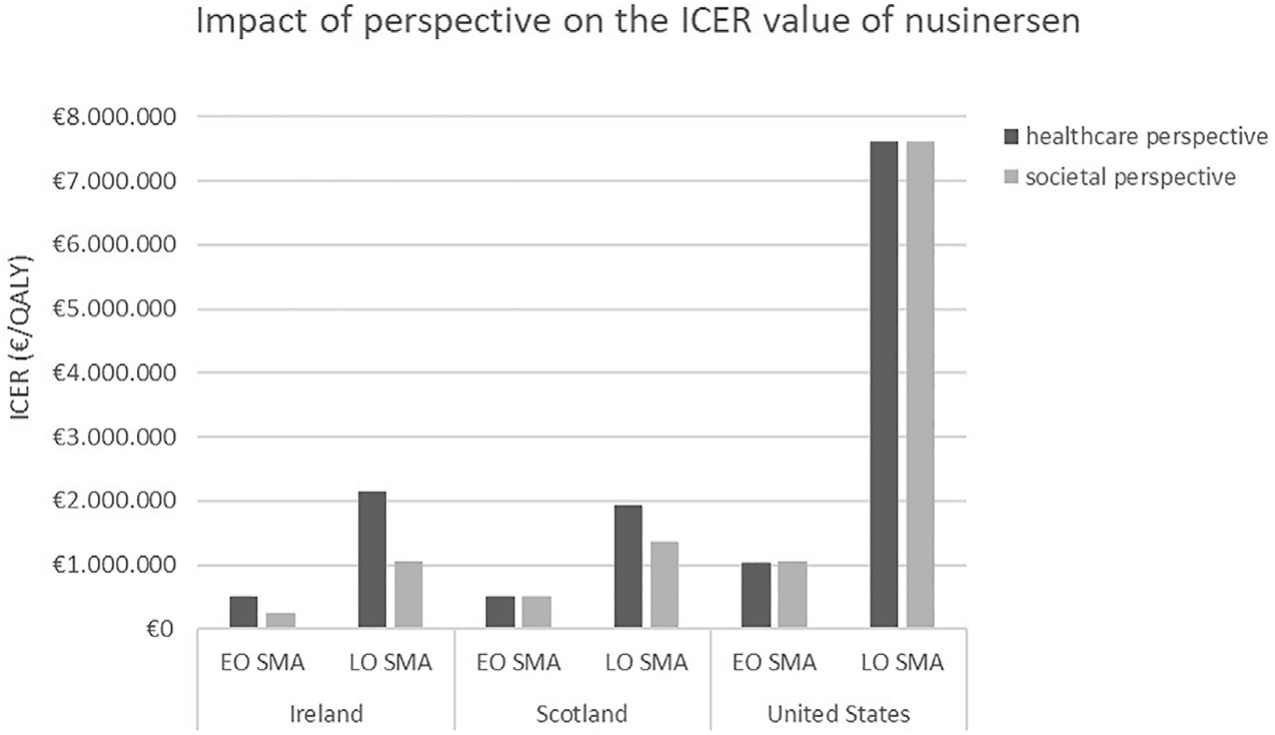
ICER values of nusinersen for EO and LO SMA, depending on the chosen perspective, reported by HTA agencies in Ireland, Scotland, and the US. Abbreviations: ICER, incremental cost-effectiveness ratio; EO, early-onset; LO, later-onset; SMA, spinal muscular atrophy.

Economic evaluations submitted to HTA agencies in France, England and Wales, Canada, Ireland, Scotland, and the US presented base case results from a healthcare payer perspective, whereas those from Sweden and the Netherlands adopted a broader, societal perspective. Additionally, in Ireland, Scotland, and the US, a scenario or secondary analysis was performed in which a societal perspective was adopted. In Ireland and Scotland, the societal perspective included costs for caregivers. The decrease of the ICER was most significant in Ireland, where shifting from a healthcare to a societal perspective lead to a decrease of approximately 50%. In the US, a (modified) societal perspective included direct nonmedical costs (such as costs for moving or modifying the patient's home and for purchasing or modifying a vehicle) and productivity gains for patients. Here, the ICER was the same for both perspectives. In the societal perspective, total costs increased similarly for patients treated with either nusinersen or standard of care, while QALY gains remained the same in both groups (see Figure 1).

Nearly all HTA agencies highlighted uncertainties regarding utilities and ICER values. These resulted from a lack of data on QoL, in addition to several shortcomings regarding the methodologies used to obtain utilities, through either PedsQL data mapping or a vignette study. Moreover, in Scotland, Canada, the US, the Netherlands, and England and Wales, Biogen’s assumptions on disease progression and hence long-term outcomes and survival rates with nusinersen were considered to be rather optimistic, especially compared to the assumptions made for the comparator group.

Only reports from England and Wales, France, and the Netherlands presented a tornado diagram, illustrating the ICERs sensitivity to mostly the vial price and utility estimates. The reports from the Netherlands, England, Wales, and Canada each reported a 0% chance for nusinersen to be cost-effective at the local willingness-to-pay threshold, whereas HAS-CEESP (France) estimated that nusinersen would have an 80% chance of being cost-effective at a threshold of €3.13 million per QALY.

#### Budget Impact Analysis

Only Belgium the Netherlands and Ireland reported the total budget impact of reimbursing nusinersen. For each of these countries, a time horizon of, respectively, three and 5 years was chosen. Whereas the Netherlands provided budget impact data for both nusinersen alone and for nusinersen combined with administration costs, the cost components were not specified in the reports issued by the HTA bodies in the remaining countries. Sweden, Germany, the Netherlands and Canada provided the estimated cost per patient per year. None of the reports mentioned sources of data for cost and patient estimates. Overall, HTA bodies cited uncertainties or a potential underestimation regarding the number of patients eligible for nusinersen treatment (Sweden, Belgium, Germany, France, and the Netherlands). Moreover, since nusinersen aims to increase life expectancy in SMA patients, patient numbers are expected to rise in the future.

#### Reimbursement Decision

Despite economic evaluations indicating that nusinersen was generally not cost-effective and despite limited data on budget impact, all countries under study reimbursed nusinersen. Germany, Belgium, and Italy provided the broadest access, by reimbursing nusinersen for all SMA patients, in line with the European label. On the other hand, access in Scotland was most narrow, reimbursing nusinersen only for EO (type I) SMA patients. In the US, access depends on the patient’s health insurance provider. The remaining countries provided access to type I, II, and III SMA patients, either with or without age restrictions. In England and Wales, additionally, presymptomatic patients were covered.

In Ireland, Scotland, Sweden, England and Wales, Belgium, and the Netherlands, reimbursement was granted under the conditions of an MEA. The MEAs in the Netherlands and Belgium are believed to be both financial and outcome-based, whereas, in England and Wales, a CED agreement was made. Overall, little information on these MEAs was available, due to the confidentiality of these agreements and their content.

## Discussion

The goal of this study was to analyze how the economic evaluation and budget impact of nusinersen in selected European jurisdictions influenced its reimbursement. We believe that our results contribute to a better understanding of the efficiency and shortcomings of the HTA in the context of orphan drugs.

### Barriers Towards the Economic Evaluation of Nusinersen

The results show that the amount and level of detail of cost-effectiveness data on nusinersen, which was either submitted by Biogen or presented by the HTA agencies, differed highly between jurisdictions. Yet, the reports described similar methodological barriers that may have complicated a proper evaluation and reassessment of the cost-effectiveness of nusinersen from the submitted models. We believe that these barriers are in alignment with those encountered for orphan drugs in general, which are extensively described in the peer-reviewed literature (Lagakos, 2003; McCabe et al., 2006; Drummond et al., 2007; Hughes-Wilson et al., 2012; Augustine et al., 2013; Schlander et al., 2016; Pearson et al., 2018; Nestler-Parr et al., 2018; Nicod et al., 2019; Blonda et al., 2021).

First of all, the HTA and reimbursement agencies reported uncertainties in utility values and questioned the added value of nusinersen in SMA subtypes, in type 0, type IV, and even type III SMA. In the past, methodologies to determine utility values have been criticized for being ill-adapted to the needs of younger patients (Eiser and Morse, 2001; Matza et al., 2004; Prosser, 2009; Ungar, 2011; Lim et al., 2014; Montgomery and Kusel, 2016; Gissen et al., 2021). These shortcomings become more impactful considering the fact that 69.9% of rare diseases present themselves in a mainly young patient population, such as in the case of SMA (Nguengang Wakap et al., 2020).

In addition, cost-effectiveness calculations mainly relied on clinical trial data, which included a healthier subgroup of SMA patients, whereas in real life, rare disease patients such as those suffering from SMA represent a heterogenous patient group, who may suffer from various comorbidities such as scoliosis and mental health issues. This heterogeneity complicates the extrapolation of treatment effects of nusinersen on the clinical trial population, to the broader, real-world patient group (Morel et al., 2013; Nicod, 2017).

Also, due to the severity of SMA, the EMA and FDA halted clinical trials early following strong interim results (EMA, 2021; Center for Drug Evaluation and Research, 2016). Indeed, clinical trials for orphan drugs are often stopped early due to a sense of urgency to market therapies for severe rare diseases (Simoens, 2011). However, this also puts an early stop to the collection of data, resulting in companies having to make assumptions as they extrapolate intermediary data to estimate the effectiveness over a horizon of 10–40 or even 80 years when submitting their reimbursement files. Indeed, we found that Biogen’s assumptions on the long-term effectiveness of nusinersen were rather optimistic compared to those of the reimbursement and HTA bodies. Unfortunately, this has further contributed to the uncertainty surrounding cost-effectiveness and budget impact estimates. In addition, this may lead to reimbursement agencies coming to a different conclusion than the regulatory agencies (EMA and FDA) (Center for Drug Evaluation and Research, 2016; EMA, 2021). In fact, for conditionally approved medicines such as nusinersen, differences in evidentiary requirements are claimed to be the main cause for the disparity between the central marketing authorization process on the one hand and the decentralized reimbursement processes on the other (Wang et al., 2018). In these cases, drug developers should initiate an early dialogue of evidentiary requirements and postlicensing commitments with both regulatory and reimbursement agencies (which, since recently, may be facilitated by EUnetHTA) in order to receive a joint scientific advice (EMA and EUnetHTA, 2021). However, we found no evidence that such efforts were made in the case of nusinersen.

Still, there is a need to streamline both the authorization and reimbursement process, as discrepancies may often lead to a duplication of the clinical assessment conducted by the HTA agency, result in delays in market entry, and contribute to uncertainty regarding patient access (Hawlik et al., 2018). This is especially the case for drugs fulfilling an unmet need such as nusinersen. These drugs may receive conditional marketing authorization from a regulatory authority, only to have their reimbursement delayed when the HTA agency does not grant them the same flexibility. Since 2018, the EU Council, together with the Member States have been developing a *Proposal for a Regulation on Health Technology Assessment (HTA) and Amending Directive 2011/24/EU* (the latter being known as the *Cross-Border Healthcare Directive*). This proposal includes the provision of a joint clinical assessment at the time of marketing authorization which, following a nonduplication principle, would mean that evidence submitted in the context of the joint assessment would not be requested again at the level of the Member States. Additionally, the proposal would support joint scientific consultations, allowing drug developers to seek early advice from HTA agencies (European Commission, 2018). Member States finally came to an agreement in March 2021 and are now expected to start negotiations with the European Parliament on a final legislative proposal (European Council, 2021). If legislation is adopted later in 2021, the first joint scientific HTA reports are to be expected in 2024 (IHS Markit, 2021).

Finally, jurisdictions highlighted the lack of local data on cost and patient numbers. We believe that herein lies an opportunity for a joint effort between authorities and the European Reference Networks (ERN). These virtual networks, formed by healthcare providers across Europe, receive funding for activities relating to research and data collection, in order to advance knowledge on rare diseases. However, many barriers are currently limiting them to reach their full potential in supporting data collection and sharing. For example, variabilities in Member State’s legal frameworks raise issues with data protection and, as such, may inhibit efficient data sharing between the Member States. Furthermore, the ERNs lack national funding for services such as maintenance of IT infrastructure dedicated to the collection of data in ERN registries. Meanwhile, the ERNs own regulations on avoiding conflicts of interest limit them from collaborating with industries or participating in research when this is entirely or partially industry-funded (Héon-Klin, 2017; Tumiene et al., 2021). We urge the Member States, together with the ERNs, to develop sustainable and efficient strategies for both internal and external collaboration. More importantly, they should develop a clear and long-term vision towards the collection and management of real-world data for rare diseases and clearly define each of the stakeholder’s role therein. We believe that this way, the ERNs may realize their full potential in their pan-European efforts to address the unmet needs of rare disease patients.

### Limitations of the Budget Impact Analyses

In general, the reports contained little details on the scope and outcome of the budget impact analysis. In the absence of data and, hence, transparency on the budget impact, questions arise regarding the quality of the analysis. For instance, when reporting on the scope of costs (included in the budget impact analysis), the analysis should consider not only drug costs but also costs for drug administration and adverse events. None of the reports clearly defined which costs were included in the analysis. Also, it is advised that a qualitative budget impact analysis includes some testing of assumptions on the estimated target population. From the available information, we found that these assumptions were only tested in the Netherlands. Finally, none of the reports disclosed data sources for either costs or target population estimated, and it is unclear whether budget impact analyses were validated. This accords with recent findings of Abdallah et al. (2021), who concluded that budget impact analyses on orphan drugs are currently of poor quality and do not fully adhere to guidelines of good practice as set up by the International Society for Pharmacoeconomics and Outcomes Research (ISPOR).

### Implicit Decision-Making Determinants and Their Impact on the Final Reimbursement Decision

The differences in methodology and reporting on the cost-effectiveness and budget impact inhibit a proper comparison of the outcomes between the jurisdictions. Such a comparison is further complicated by the fact that countries have implemented different value assessment frameworks, some of which were adapted specifically to allow more flexibility for orphan drugs, such as in Ireland, Scotland, and England and Wales, while others are catered to treatments indicated for a severe disease, such as in Sweden and the Netherlands (Stolk et al., 2004; SMC, 2012; van de Wetering et al., 2013; van de Wetering et al., 2015; Kanters, 2016; NICE, 2017; Pearson et al., 2018; Nicod and Whittal, 2020). Nevertheless, there appears to be no correlation between nusinersen’s cost-effectiveness and the SMA subtypes for which reimbursement was granted. The countries that highlighted weaknesses or scarcity of effectiveness data in either type 0, type IV, or type III SMA still provided reimbursement for patients with these indications, with the exception of Scotland.

**FIGURE 2:**
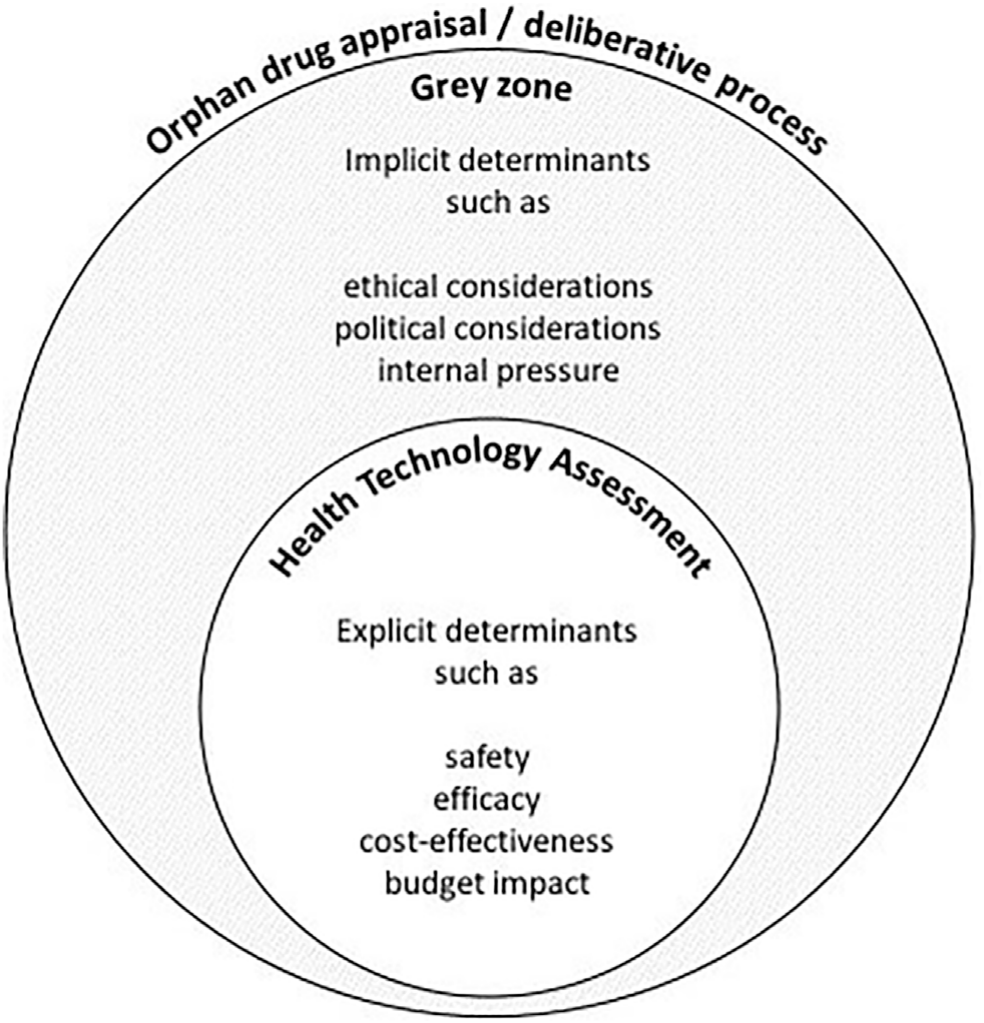
A schematic and nonexhaustive visualization of the deliberative process for the reimbursement of an orphan drug such as nusinersen. Any discrepancy between the assessment and appraisal of an orphan drug may be the result of grey zone determinants that are not adequately described in the final reimbursement report.

The fact that nusinersen was reimbursed despite reportedly uncertain or unfavorable ICER values and budget impact implies that decision-makers considered other implicit determinants that favored a positive reimbursement decision. This suggests that there exists a grey zone between the assessment and (final) appraisal step of the reimbursement process. Whereas the assessment is detailed in the HTA report, which describes the orphan drug’s performance against mainly clinical (safety and efficacy) and economic (cost-effectiveness and budget impact) criteria, the reports generally do not elaborate on the discussion that took place during the appraisal step. The implicit determinants, hereafter referred to as “grey zone” determinants, which play a role during the appraisal remain vague, and thus, it can be argued that the final decision is poorly substantiated (see Figure 2).

For instance, in several countries, political factors and/or pressure from media outlets and disease advocacy organizations may have contributed to a positive reimbursement decision. According to King and Bishop, the “hype” that these stakeholders created around nusinersen may have resulted in an overestimation of the beneficial treatment effects of nusinersen, while the risks were minimized. Moreover, while access to public information grows, it becomes increasingly difficult to admit that certain uncertainties concerning cost and effectiveness data have remained. In addition, a close relationship between the patient organization CureSMA and the nusinersen research team may have skewed information and data (King and Bishop, 2017). The influence of patient organizations was also prominent in Ireland, where SMA Ireland launched a petition after the national health authority, Health Service Executive (HSE), and Biogen failed to come to an agreement on the reimbursed price of nusinersen. Nearly 4 months later, following an intense campaign by both SMA patients and their family members, the HSE granted reimbursement for nusinersen for type I, II, and III SMA patients (Ryan, 2019a; Ryan, 2019b; Ryan, 2021).

As mentioned before, many countries are adapting their standard processes to tailor to the needs of orphan drugs. For instance, in Scotland, reimbursement was initially granted only for type I SMA patients, while type II and III SMA patients were excluded. However, shortly after the initial appraisal, a reimbursement pathway for ultra-orphan drugs was implemented that allowed a broader conditional reimbursement for type II and III SMA patients for a period of 3 years, after which nusinersen will be reassessed based on additional evidence (scheduled for 2022) (SMC, 2021a). However, this “revised” reimbursement decision for type II and III SMA was based on the original HTA report. This implies that during the reassessment different reimbursement criteria were applied or that at least more leniency was granted for nusinersen in types II and III compared to type I SMA. Unfortunately, the arguments in favor of broadening the reimbursement of nusinersen were not publicly specified.

Additionally, we have reason to assume that ethical arguments played a role in the decision-making process. For instance, in its final advice, ZIN underlined the solidarity principle, while both the SMC (Scotland) and ZIN (the Netherlands) mentioned unmet medical need as an important argument in favor of reimbursement. In Scotland, unmet medical need is formally included as a decision modifier, which may allow a higher ICER threshold compared to standard treatments (SMC, 2012). In England and Wales, the report stated that the rarity and severity of SMA were considered, although it did not disclose the extent to which these factors influenced the final decision (NICE, 2019). Meanwhile, TLV concluded its report with a statement that, in general, drug decision-making is based on three principles: 1) human value, 2) need and solidarity, and 3) cost-effectiveness. However, it did not mention to what extent it found that nusinersen met either of these three principles (NT-council, 2019).

Indeed, the fact that decision-makers are increasingly balancing efficiency criteria (such as cost-effectiveness and budget impact) with ethical criteria (such as severity or unmet need), when assessing the value of orphan drugs, is not new (Pinxten et al., 2012; Nicod et al., 2017; Burgart et al., 2018; Nicod et al., 2019; Blonda et al., 2021). However, there is a need for more transparency on the factors that influence decision-making after an HTA guidance is issued, as this is becoming increasingly important in order to substantiate decisions on budget allocation, especially in those cases where there is substantial uncertainty on the cost and/or effectiveness of treatment. Additionally, by disclosing the grey zone determinants, decision-makers facilitate a broader acceptance of the reimbursement decision among the different stakeholders (Youngkong et al., 2012; Iskrov et al., 2017; Schey et al., 2017; Baltussen et al., 2018; Bond et al., 2020; Blonda et al., 2021). In order to increase transparency on the appraisal process, we therefore advise decision-makers to define and formally include these determinants in the reimbursement process and the final reimbursement report. In fact, next to “transparency,” the principle of “inclusivity” and “impartiality” make up the three core principles around which Bond et al. (2020) propose to structure the appraisal or deliberative process of a health technology. The principle of inclusivity aims to ensure that all stakeholders, including patient representatives, are represented during the decision-making process and that their views are genuinely considered. The impartiality principle aims towards a process that is free from both internal and external influences. This could be done, for instance, by describing how a campaign and petition by a patient advocacy organization such as SMA Ireland may have shifted the opinions of the stakeholders involved in decision-making. By structuring the appraisal process according to these three core principles, we believe that decision-makers can minimize the influence of external pressure and/or political considerations on decision-making, especially in the case of innovative yet expensive orphan drugs such as nusinersen.

### Managed Entry Agreements as a Tool to Manage Uncertainty

We assume that the outcomes of the cost-effectiveness and budget impact analyses included in the HTA report, together with the grey zone determinants, act as a facilitator and starting point for setting up MEAs. These agreements have become a tool that enables decision-makers to allow reimbursement, albeit conditional, while managing the remaining uncertainties surrounding cost and effectiveness. Still, there are no public data on how confidential rebates have improved the cost-effectiveness of nusinersen or on how data uncertainties are revised after the MEA period (Gerkens et al., 2017). When the real cost-effectiveness and budget impact of nusinersen cannot be assessed, there is no transparency on whether financial resources are fairly allocated across disease areas. Also, there will be no benchmarks to which future orphan drugs may be compared. Still, decision-makers will face similar barriers when deciding on future therapies for rare diseases, such as Risdiplam® and the gene-therapy Zolgensma® for SMA, which are potentially curative but will have a considerably higher price. Providing a high and unjustified price to these innovative drugs may boost the price of future treatments even more, especially those for other rare conditions that are still lacking an adequate reference treatment. In addition, increasing expenditure on orphan drugs risks to disrupt healthcare systems worldwide, especially in low- and middle-income countries, and may further contribute to unequal access for SMA patients.

### Study Limitations

Our study is not without limitations. First of all, reporting on budget impact was incomplete and nontransparent, which made it difficult to draw conclusions on its use in decision-making. Second, we analyzed reimbursement reports from a limited number of jurisdictions, which may have led to selection bias. The inclusion of additional jurisdictions could have led to different conclusions. However, as we selected jurisdictions that were spread throughout the EU, we believe that our findings are generalizable to orphan drugs in general. Third, by using an online translator such as Google Translate, there is a potential risk of misinterpretation of data due to translation errors. Fourth, more thorough information on MEAs could have been retrieved by performing a systematic literature review, including search terms in languages from all respective countries.

## Conclusion

This study has analyzed how the economic evaluation and budget impact of nusinersen in selected European jurisdictions influenced its reimbursement. Furthermore, it has contributed to a better understanding of the role of economic criteria on the reimbursement of orphan drugs in general.

The results confirmed that an HTA based on economic criteria alone is not sufficient to define the value of orphan drugs. However, by not being transparent on the “grey zone determinants” in favor of reimbursement, the economic evaluation loses its value as a tool to effectively rank orphan drugs and allocate funds from a limited budget. We suggest that decision-makers provide more transparency on the appraisal process of orphan drugs and on the requirements that are negotiated in the context of an MEA. By formally incorporating all determinants into a reimbursement process that is transparent, inclusive, and impartial, decision-makers contribute to a sustainable environment for orphan drugs and future therapies.
